# Validation of the Arabic Linguistic Version of the Prolapse and Incontinence Knowledge Questionnaire

**DOI:** 10.1007/s00192-024-05823-2

**Published:** 2024-05-31

**Authors:** Ahlam M. Al-Kharabsheh, Seham M. Abufraijeh, Nedal Al-Nawaiseh

**Affiliations:** 1 Department of Obstetrics and Gynecology, Faculty of Medicine, Mutah University, Alkarak, Jordan; 2 Department of Public Health, Faculty of Medicine, Mutah University, Alkarak, Jordan

**Keywords:** Pelvic floor dysfunction, Urinary incontinence, Pelvic organ prolapse, Knowledge assessment, Arabic Prolapse and Incontinence Knowledge Questionnaire

## Abstract

**Introduction and Hypothesis:**

Pelvic floor disorders (PFDs), significantly impacting women's quality of life, are often underdiscussed owing to misconceptions and limited understanding of treatment options. This study is aimed at validating an Arabic version of the Prolapse and Incontinence Knowledge Questionnaire (PIKQ) to assess knowledge of pelvic organ prolapse (POP) and urinary incontinence (UI) among Arabic-speaking women, addressing knowledge gaps in these areas.

**Methods:**

The study obtained ethical approval and followed a two-stage process, including a pilot study for preliminary validation and a larger study involving 300 participants. The PIKQ, a self-administered tool with two scales focusing on UI and POP, was translated into Arabic with cultural and linguistic adaptations. The study evaluated the reliability and validity of the Arabic PIKQ, employing Cronbach’s alpha, intraclass correlation coefficient (ICC), and Spearman’s rho for reliability assessments, as well as factor analysis for construct validity.

**Results:**

The Arabic PIKQ demonstrated high internal consistency (Cronbach’s alpha > 0.8) and test–retest reliability (ICC > 0.79) for both the UI and the POP scales. The questionnaire also showed significant construct validity. Among the 300 participants, knowledge gaps were evident, influenced by educational and professional backgrounds. Notably, 22% reported UI and 14.7% reported pelvic organ prolapse, with less than half seeking treatment.

**Conclusions:**

The Arabic PIKQ has been validated as a reliable tool for improving knowledge and addressing misconceptions regarding PFDs among Arabic-speaking women. The study underscores the importance of culturally sensitive educational tools in enhancing awareness and facilitating access to medical care for pelvic floor disorders.

## Introduction

Female pelvic floor dysfunction (PFD) is characterized by a diverse array of symptoms and anatomical alterations arising from abnormalities in the functioning of the pelvic floor muscles [1, 2]. The most prevalent PFD disorders are urinary incontinence (UI), pelvic organ prolapse (POP), and fecal incontinence.

It is observed that women tend to be reluctant to initiate discussions concerning their urinary complaints or symptoms associated with prolapse. This reluctance can be attributed to a limited understanding of the various treatment options available in both domains and the misconception that surgery is the sole solution [3–5].

Studies have shown that pelvic floor disorders can have a substantial impact on the quality of life of affected individuals, often resulting in debilitating conditions [6, 7]. As a result, it is crucial to promote and facilitate women's access to medical assistance for these issues. Achieving this goal requires the enhancement of awareness, which can be accomplished by evaluating women's levels of knowledge and addressing misconceptions regarding risk factors, diagnosis, and treatment options.

In evaluating community knowledge and attitudes toward a particular disease, the cultural adaptation of assessment tools, such as questionnaires, is crucial. This adaptation involves tailoring aspects such as linguistic usage and content relevance to the specific cultural milieu of the target population, thereby enhancing the tool's applicability and effectiveness. There are two primary methodologies for this purpose:

1. The development of a novel measurement tool tailored to the specific context.
2. The utilization of an existing instrument initially developed in a different language, with appropriate modifications for cultural and linguistic congruency [8].

The primary objective of this study is to present a validated Arabic version of the Prolapse and Incontinence Knowledge questionnaire (PIKQ), a measure known for its reliability and validity in assessing knowledge related to POP and UI [9]. The Arabic version holds significant value for utilization in Arabic-speaking regions. A secondary goal is to assess the extent of knowledge concerning pelvic floor disorders, with a particular focus on POP and UI, and subsequently, to address and reduce the existing knowledge gaps pertaining to these conditions.

## Materials and Methods

For this study, ethical approval was granted by an institutional Ethics Committee, as indicated by the reference number [1422024], in accordance with the Declaration of Helsinki (as revised in 2013). The research was conducted in two distinct stages, after obtaining permission from the authors of the English version of the PIKQ [9].

### Translation

The PIKQ, a validated tool intended for self-administration, is aimed at evaluating patient knowledge through two distinct scales, each consisting of 12 items. These scales focus on topics related to UI and POP, covering areas such as epidemiology, diagnosis, and treatment modalities.

**Fig. 1:**
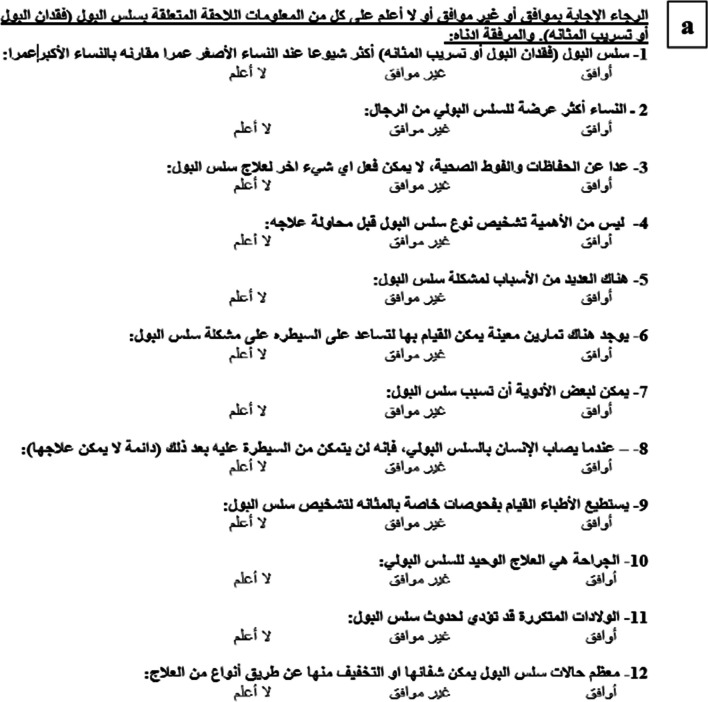

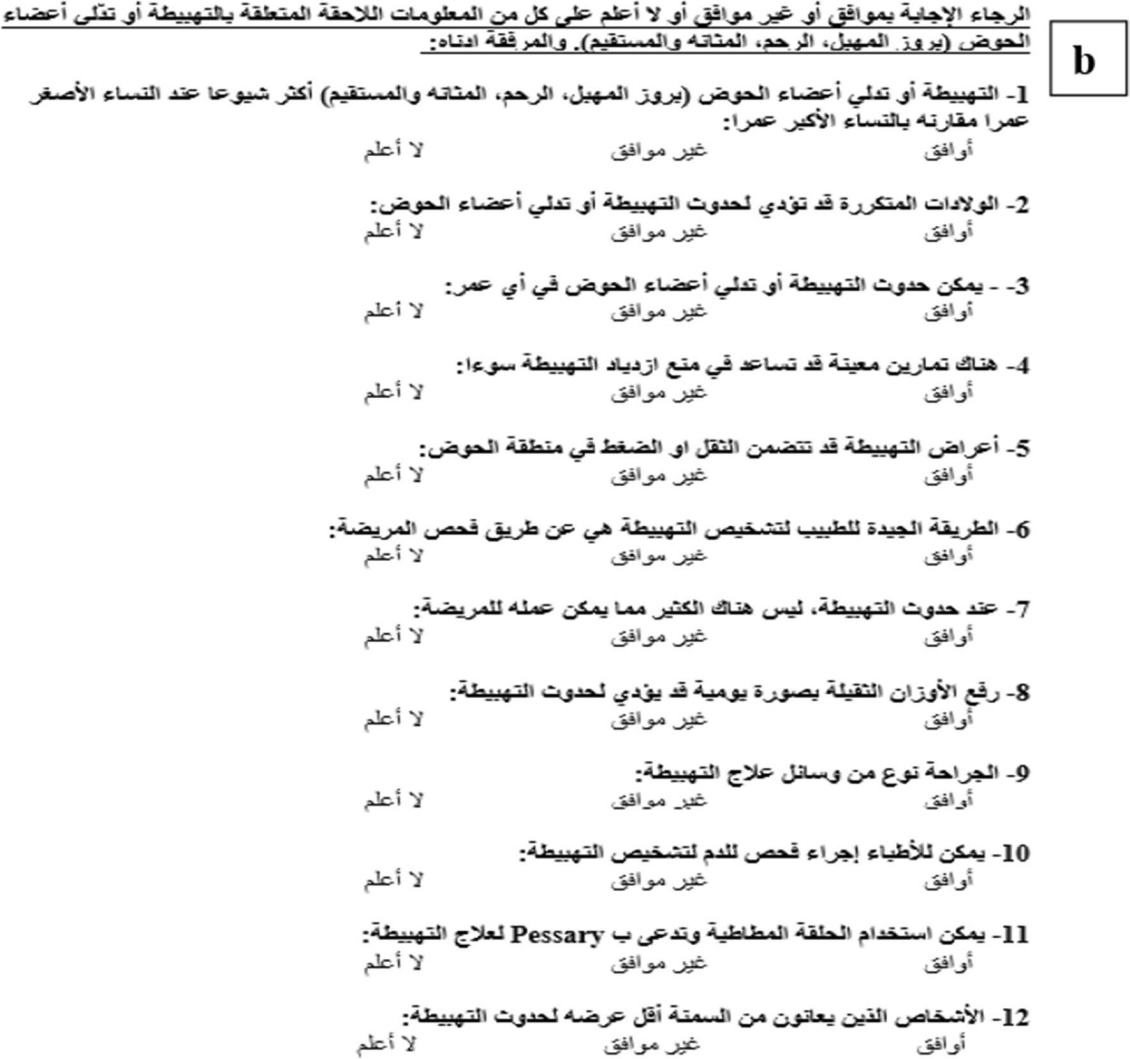

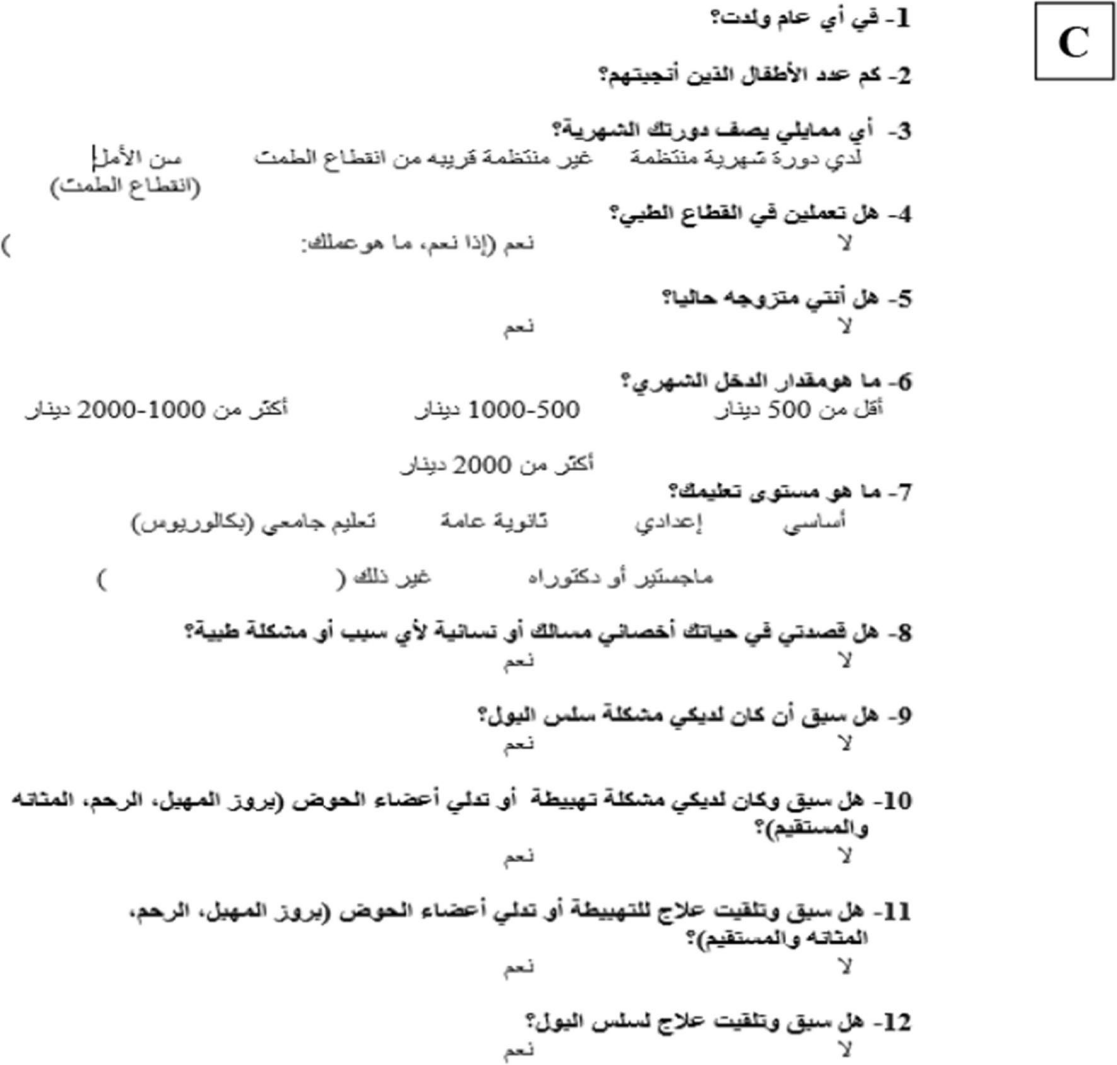
**a** Arabic Prolapse and Incontinence Knowledge Quiz (PIKQ) Urinary Incontinence scale. **b** Arabic PIKQ Pelvic Organ Prolapse scale. **c** Demographic questions

In the process of adapting the PIKQ for our population, modifications were made in the third section of the questionnaire, dedicated to demographic data (Fig. 1c). Specifically, question 6 was altered from assessing annual household income in US dollars to evaluating monthly income in Jordanian Dinar. Furthermore, question 13 was removed owing to its irrelevance in the context of this study.

The translation of the PIKQ from English into Arabic, the intended language, was conducted by two gynecologists. Among them was a native speaker of Arabic who is also bilingual in English. Additionally, a urogynecology expert contributed to the translation, resulting in the creation of three distinct Arabic adaptations of the questionnaire. Each translation was conducted independently. To ensure uniformity and accuracy, a consensus meeting was organized where the team discussed and resolved any discrepancies and established a unified Arabic version (Fig. 1a, b).

To verify the precision of the translation, the Arabic version was retranslated back into English by two linguists. These individuals were skilled in English as their first foreign language and had no previous familiarity with the PIKQ, lacking a medical background. A comparative examination was then performed between the original PIKQ and its English retranslation. Discrepancies identified in this comparison were thoroughly addressed and resolved in a revision meeting that involved all members of the translation team.

### Participants

Participants for this study were recruited from the obstetrics and gynecology unit of a tertiary-care government hospital in the southern region of Jordan, during the period June 2023 to December 2023. Inclusion criteria encompassed women over the age of 18 who visited the hospital and were willing to participate in the study. Although the questionnaire was designed for self-administration, women who were unable to read and expressed a willingness to participate were provided with non-intrusive assistance from a research assistant.

Exclusion criteria for the study were age younger than 18 years, pregnancy, inadequate cooperation, or submission of incomplete questionnaire responses.

Before commencing the questionnaire, all enrolled participants provided informed consent by signing a written consent form. This form was accompanied by a cover letter that comprehensively outlined the study's objectives and assured participants of the strict confidentiality measures in place to safeguard their provided data.

In addition to the questionnaire assessing knowledge related to UI and POP (Fig. 1a, b), participants were presented with a second component comprising demographic questions (Fig. 1c). These demographic questions were also translated and included inquiries about participants' age, employment status, educational background, marital status, menstrual history, and the number of childbirths. Additionally, binary “yes” or “no” questions were included to identify any reported issues related to urinary leakage, POP, or prior treatments for these conditions. Participants were required to complete both components in their entirety.

Body weight and height measurements were taken during the interview sessions, and the calculation of body mass index (BMI) was based on these measurements obtained at the time of the examination. BMI classifications were defined as follows: underweight (< 18.5 kg/m^2^), normal weight (18.5–24.9 kg/m^2^), overweight (25.0–29.9 kg/m^2^), and obese (≥ 30 kg/m^2^) [10].

### Validation Study

The translated questionnaire was administered to a cohort of 32 women. Each participant provided written consent prior to participation. They were informed that a follow-up session would be conducted 2 weeks later for repeat administration of the questionnaire. This preliminary investigation was designed to evaluate the content validity of the questionnaire and to refine it to its final form. This refinement process involved conducting interviews with participants to discuss any ambiguous questions or difficulties encountered with the questionnaire.

Furthermore, the pilot study facilitated the assessment of the internal consistency of the questionnaire, examining the interrelationship among its items. It also enabled the evaluation of test–retest reliability, assessing the stability of responses provided by individuals across the two administrations of the questionnaire.

In the formal comparative study, 300 women were enrolled after obtaining informed consent with the same inclusion criteria for those recruited in the pilot study.

### Assessment

In each scale of the questionnaire, a scoring mechanism was implemented where correct answers were assigned a value of 1, whereas “do not know”, incorrect, or unprovided responses were assigned a value of 0. The total scores for the UI and POP scales were computed by summing the correct responses, yielding a score range from 0 to 12. A higher score is indicative of a greater knowledge level regarding UI and POP (Fig. 1a, b).

The psychometric properties of the UI and POP scales were rigorously evaluated. This evaluation encompassed an analysis of scale validity, internal consistency, and test–retest reliability. To quantify internal consistency, Cronbach’s alpha coefficient was utilized for each scale. Furthermore, to determine test–retest reliability, the PIKQ was re-administered to a subset of 32 participants from the pilot cohort, 2 weeks after their initial questionnaire completion.

### Statistical Analysis

Statistical analysis in this study employed IBM SPSS Statistics 25.0 (2017; IBM, Armonk, NY, USA) for descriptive statistics, including mean, standard deviation, frequencies, and percentages. Psychometric properties of knowledge scales were assessed through reliability analysis, specifically Cronbach's alpha coefficient for internal consistency. Test–retest reliability was assessed using the intraclass correlation coefficient (ICC) and 95% confidence intervals (CI). Pearson's Chi-squared test and Fisher’s exact test examined the relationships between categorical variables and outcomes, with significance set at *p* ≤ 0.05. The Kaiser–Meyer–Olkin (KMO) test assessed sample sufficiency and multivariate normality. Bartlett's test of sphericity evaluated sample suitability for factor analysis to assess construct validity. Factor analysis was performed using principal component analysis with varimax rotation.

In line with established literature guidelines, the determination of the study's sample size followed the recommendation of a minimum of 10 participants per parameter for confirmatory factor analysis [11–13]. Initially, an estimation of 240 subjects was made, considering PIKQ with its 24-item survey, including two 12-item subscales. However, to account for potential dropouts or data collection losses, the sample size was subsequently increased.

Ordinal logistic regression was applied to identify the contribution of demographic and clinical variables and information-seeking preferences to the knowledge score.

## Results: Pilot Study Insights

In a preliminary pilot study 32 women participated.

### Reliability and Validity Assessments

The internal consistency of the questionnaire was robust, evidenced by Cronbach’s alpha coefficients of 0.824 for the UI score and 0.811 for the POP score. Test–retest reliability was assessed using the ICC, demonstrating high reliability, with values of 0.828 for the UI scale and 0.798 for the POP scale. Spearman’s rho coefficient further confirmed reliability, yielding correlations of 0.834 for the UI scale and 0.884 for the POP scale, with a significance level for both *p* < 0.01 (Table 1).

**Table 1:** Reliability and test scores for urinary incontinence (*UI*) and pelvic organ prolapse (*POP*) scales in the pilot study

Variable description	UI scale	POP scale
Cronbach’s alpha	0.824	0.811
Intraclass correlation	0.828 (95% CI: 0.751 to 0.889)	0.798 (95% CI: 0.708 to 0.870)
Spearman’s rho	0.834 (*p* < 0.01)	0.884 (*p* < 0.01)
Test score mean ± SD (initial)	10.09 ± 2.704 (*p* = 0.006)	9.19 ± 2.956 (*p* = 0.019)
Test score mean ± SD (retest)	10.31 ± 2.402	9.44 ± 2.651

*SD* standard deviation

*p* values indicate the significance level of the test–retest mean differences

The ICC for the PIKQ-UI was 0.828, with a 95% CI from 0.751 to 0.889, and for the PIKQ-POP, it was 0.798 (95% CI: 0.708 to 0.870). Both *p* values were less than 0.001, indicating strong consistency. The mean test scores for PIKQ-UI remained consistent between initial testing (10.09 ± 2.704) and retesting (10.31 ± 2.402), whereas PIKQ-POP scores increased slightly from 9.19 ± 2.956 to 9.44 ± 2.651, with significant *p* values of 0.006 and 0.019 respectively (Table 1).

### Study Background and Participant Demographics

In the validation study of the Arabic version of the Prolapse and Incontinence Knowledge Questionnaire, 300 patients participated. The median age of these participants was 43 years, ranging from 18 to 75 years. Notably, the median BMI was 29 kg/m^2^, with approximately 46% classified as obese. A significant subset (6.7%, *n* = 20) were medical professionals, and 35% held a bachelor's degree or higher. Most participants (81.7%) were in a marital relationship. Among the married women (*n* = 245), 7.8% were nulliparous, and 47.3% were grand multipara. Within this cohort, 22% reported experiencing UI issues, and 14.7% reported POP. Treatment was received by 37.9% (25 out of 66) of those with UI, whereas 40.9% (18 out of 44) of patients with prolapse-related symptoms underwent treatment, as indicated in Table 2.

**Table 2:** Basic characteristics of study participants

Characteristic	Data
Age, years, mean, SD (*N* = 300)	42.9 ± 11.7
19–25, *n* (%)	28 (9.3)
26–35, *n* (%)	58 (19.3)
36–45, *n* (%)	99 (33)
46–55, *n* (%)	71 (23.7)
56–65, *n* (%)	37 (12.3)
>65, *n* (%)	7 (2.3)
Parity, *n* (%), *N* = 245	
Nulliparous	19 (7.8)
1–4	110 (44.9)
≥ 5	116 (47.3)
BMI (kg/m^2^), *n* (%)	
18–24.9	44 (14.7)
25–29.9	116 (38.7)
30–34.9	76 (25.3)
>35	64 (21.3)
Education level, *n* (%)	
Illiterate	37 (12.3)
Elementary	36 (12)
Secondary school	122 (40.7)
Bachelor	98 (32.7)
High degree	7 (2.3)
Hormonal status, *n* (%)	
Regular cycles	193 (64.3)
Premenopausal	36 (12)
Postmenopausal	70 (23.3)
Marriage status, *n* (%)	
Yes	245 (81.7)
No	55 (18.3)
Medical field, *n* (%)	
Yes	20 (6.7)
No	280 (93.3)
Income, (Jordanian Dinar) *n* (%)	
< 500	233 (77.7)
500–1,000	58 (19.3)
> 1,000	9 (3)
Ever had urine incontinence, *n* (%)	
Yes	66 (22)
No	234 (78)
Ever had pelvic organ prolapse, *n* (%)	
Yes	44 (14.7)
No	256 (85.3)
Ever been treated for urine incontinence, *n* (%), *N* = 66	
Yes	25 (37.9)
No	41 62.1)
Ever been treated for pelvic organ prolapse, *n* (%), *n* = 44	
Yes	18 (40.9)
No	26 (59)
Ever sought urology/urogynecology help, *n* (%)	
Yes	228 (76)
No	72 (24)

*SD* standard deviation, *BMI* body mass index

### Factor Analysis and Construct Validity

The construct validity was thoroughly assessed.

The KMO measure yielded coefficients of 0.637 for the UI domain and 0.641 for the POP domain, indicating a moderate suitability for factor analysis in both domains. Additionally, Bartlett's test of sphericity produced significant results with Chi-squared (66) = 197.316 (*p* < 0.001) for the UI domain and Chi-squared (66) = 183.545 (*p* < 0.001) for the POP domain, further confirming the appropriateness of the dataset for factor analysis.

The establishment of construct validity was achieved through a principal component analysis, employing an extraction method alongside varimax rotation. Within this framework, the PIKQ-UI revealed a structure encompassing four factors, characterized by eigenvalues between 1.04 and 2.009. This structure accounted for 46.77% of the total variance. The range of factor loadings for this component ranged from 0.352 (item 11) to 0.766 (item 12). On the other hand, the PIKQ-POP exhibited a five-factor structure, with eigenvalues ranging from 1.024 to 2.016 and accounting for 54.173% of the total variance. The loadings for these factors varied, with the lowest being 0.478 (item 1) and the highest reaching 0.835 (item 12), as detailed in Table 3.

**Table 3:** Rotated factor loadings of items from each of the two domains of the Arabic Prolapse and Incontinence Knowledge Questionnaire

Factor	Item		Factor load
PIKQ-UI: factor loads after varimax rotation (*n* = 300)			
1	1	Urinary incontinence is more common in young women than in old women	0.510
	2	Women are more likely than men to leak urine	0.516
	3	Other than pads and diapers, not much can be done to treat leakage of urine	0.620
	4	It is not important to diagnose the type of urine leakage before trying to treat it	0.601
	8	Once people start to leak urine, they are never able to control their urine again	0.597
2	5	Many things can cause urinary leakage	0.626
	6	Certain exercises can be done to help control urine leakage	0.533
	9	Doctors can do special types of bladder testing to diagnose urine leakage	0.634
	11	Giving birth many times may lead to urine leakage	0.352
3	7	Some medications may cause urinary leakage	0.645
	10	Surgery is the only treatment for urinary leakage	0.671
4	12	Most people who leak urine can be cured or improved with some kind of treatment	0.766
PIKQ-POP: factor loads after varimax rotation (*n* = 300)			
1	3	Pelvic organ prolapse can happen at any age	0.614
	4	Certain exercises can help to stop pelvic organ prolapse from getting worse	0.663
	8	Heavy lifting on a daily basis can lead to pelvic organ prolapse	0.619
2	1	Pelvic organ prolapse (bulging of the vagina, bladder, or rectum) is more common in young women than in old women	0.478
	6	A good way for a doctor to diagnose pelvic organ prolapse is by examining the patient	0.696
	11	A rubber ring called a pessary can be used to treat symptoms of pelvic organ prolapse	0.650
3	7	Once a patient has pelvic organ prolapse, not much can be done to help her	0.723
	10	Doctors can run a blood test to diagnose pelvic organ prolapse	0.669
4	2	Giving birth many times may lead to pelvic organ prolapse	0.496
	12	People who are obese are less likely to get pelvic organ prolapse	0.835
5	5	Symptoms of pelvic organ prolapse may include pelvic heaviness and/or pressure	0.503
	9	Surgery is one type of treatment for pelvic organ prolapse	0.813

*PIKQ-UI* Prolapse and Incontinence Knowledge Quiz Urinary Incontinence, *PIKQ-POP* Prolapse and Incontinence Knowledge Quiz Pelvic Organ Prolapse

### Score Comparisons and Analysis

On the multiple regression analysis aimed at identifying predictive factors associated with increased knowledge about UI and POP, several variables were examined (Table 4). The results indicate that individuals with an increasing parity, higher educational level, and those who have previously sought medical assistance to urology or urogynecology exhibit a statistically significant association with greater knowledge about UI (*p* values: 0.022, 0.002, and 0.005 respectively).

**Table 4:** Comparisons of Prolapse and Incontinence Knowledge Quiz Urinary Incontinence (*PIKQ-UI*) and Prolapse and Incontinence Knowledge Quiz Pelvic Organ Prolapse (*PIKQ-POP*) scores across some characteristics and predictive factors for a higher level of knowledge

Characteristics	*n*	PIKQ-UI (mean ± SD)	PIKQ-POP (mean ± SD)
Age (years)			
18–25	28	8.75 ± 2.084	7.97 ± 1.572
26–35	58	8.29 ± 1.901	7.98 ± 1.55
36–45	99	8.63 ± 2.225	8.18 ± 1.992
46–55	71	8.79 ± 1.788	8.38 ± 1.8
56–65	37	8.32 ± 1.827	8.35 ± 1.602
> 65	7	7.8 ± 2.775	8.6 ± 1.14
*p* value		0.808	0.099
Parity	245		
Nulliparous	19	8.56 ± 2.089	8.17 ± 1.792
1–4	110	8.35 ± 2.127	8.11 ± 1.731
≥ 5	116	8.76 ± 1.857	8.26 ± 1.795
*p* value		0.022*	0.899
BMI (kg/m^2^)			
18–24.9	44	8.76 ± 1.857	8.26 ± 1.795
25–29.9	116	8.59 ± 2.069	8.33 ± 1.783
30–34.9	76	8.62 ± 2.059	7.97 ± 1.804
> 35	64	8.86 ± 1.825	8.31 ± 1.851
*p* value		0.061	0.805
Income			
< 500 JD	233	8.45 ± 2.034	8.08 ± 1.681
500–1,000 JD	58	9.17 ± 1.798	8.67 ± 2.047
> 1,000 JD	9	8.14 ± 2.116	7.57 ± 1.813
*p* value		0.55	0.248
Education			
Illiterate	37	8.03 ± 1.641	8.27 ± 1.521
Elementary	36	8.56 ± 2.197	7.92 ± 1.857
Secondary school	122	8.26 ± 2.020	8 ± 1.781
Bachelor	98	9.09 ± 1.905	8.38 ± 1.732
High degree	7	9.29 ± 2.928	9.57 ± 2.225
*p* value		0.002*	0.094
Works in medical Field			
Yes	20	9.4 ± 1.875	9.1 ± 2.198
No	280	8.5 ± 2.016	8.12 ± 1.718
*p* value		0.146	0.065
Ever sought urology/urogynecology help			
Yes	228	8.76 ± 1.975	836 ± 1.751
No	72	7.94 ± 2.034	7.64 ± 1.714
*p* value		0.005*	0.009*
Ever had UI problem			
Yes	66	8.79 ± 2.00	8.41 ± 1.736
No	234	8.46 ± 2.04	8.12 ± 1.773
*p* value		0.262	0.272
Ever had prolapse problem			
Yes	44	8.45 ± 2.040	8.59 ± 1.821
No	256	8.55 ± 2.034	8.11 ± 1.751
*p* value		0.542	0.130
Ever received prolapse treatment			
Yes	18	8.44 ± 2.175	8.33 ± 1.609
No	282	8.54 ± 2.027	8.17 ± 1.778
*p* value		0.965	0.521
Ever received UI treatment			
Yes	25	8.72 ± 2.092	8.12 ± 2.027
No	275	8.55 ± 2.013	8.19 ± 1.745
*p* value		0.881	0.316

*SD* Standard deviation, *BMI* body mass index, *UI* urinary incontinence

*p value < 0.05

Furthermore, individuals who have sought medical help also tend to possess higher knowledge levels related to POP, as indicated by a *p* value of 0.009. Additionally, individuals working in the medical field and those with a higher level of education demonstrate trends towards being significant predictors of knowledge to prolapse; however, these findings do not reach statistical significance (*p* values: 0.065 and 0.094 respectively).

Importantly, none of the following factors, including age, BMI and having ever received UI treatment, ever having had prolapse treatment, or experiencing UI or prolapse complaints, show statistical significance in influencing knowledge scores for either UI or POP, as indicated by their *p* values being greater than 0.05.

### Analysis of the Knowledge Scales

Table 5 presents the item-wise and total scores for the PIKQ-UI and PIKQ-POP among the sample of 300 participants. Each item was scored on a scale, with higher scores indicating greater knowledge.

**Table 5:** Item and total scores for Prolapse and Incontinence Knowledge Quiz Urinary Incontinence (*PIKQ-UI*) and Prolapse and Incontinence Knowledge Quiz Pelvic Organ Prolapse (*PIKQ-POP*)

Items (*n* = 300)	PIKQ-UI, mean ± SD	Average correct answers (%)	PIKQ-POP, mean ± SD	Average correct answers (%)
1	0.6 ± 0.49	60	0.6 ± 0.491	60
2	0.7 ± 0.441	70	0.87 ± 0.341	87
3	0.61 ± 0.488	61	0.54 ± 0.499	54
4	0.69 ± 0.463	69	0.64 ± 0.480	64
5	0.9 ± 0.296	90	0.86 ± 0.351	86
6	0.62 ± 0.487	62	0.95 ± 0.225	95
7	0.6 ± 0.490	60	0.72 ± 0.448	72
8	0.69 ± 0.463	69	0.77 ± 0.424	77
9	0.93 ± 0.256	93	0.77 ± 0.422	77
10	0.64 ± 0.482	64	0.45 ± 0.499	45
11	0.63 ± 0.483	63	.28 ± 0.448	28
12	0.88 ± 0.326	88	0.72 ± 0.448	72
Total	8.54 ± 2.025	71	8.17 ± 1.768	68

*SD* standard deviation

For the PIKQ-UI scale, the mean item scores ranged from 0.60 (SD = 0.49) to 0.93 (SD = 0.256), translating to an average correct answer percentage ranging from 60 to 93%. Notably, item 9 (Doctors can do special types of bladder testing to diagnose urine leakage) showed that 93% of participants answered this item correctly, reflecting a high level of knowledge on this aspect. Conversely, item 1 (UI is more common in young women than in old women) had the lowest percentage of correct answers (60), suggesting a comparatively lower knowledge level. The total mean score for the PIKQ-UI scale was 8.54 (SD = 2.025), corresponding to an overall average correct answer percentage of 71.

Similarly, for the PIKQ-POP scale, item scores varied, with a range from 0.28 (SD = 0.448) for item 11 to 0.95 (SD = 0.225) for item 6. These scores indicate an average correct answer percentage from as low as 28% to as high as 95% respectively. Item 6 (A good way for a doctor to diagnose POP is by examining the patient), with the highest score, suggests a high understanding of that aspect of POP among the participants. In contrast, item 11 (A rubber ring called a pessary can be used to treat symptoms of POP), with the lowest score, highlights a significant knowledge gap. The total mean score for the PIKQ-POP scale was 8.17 (SD = 1.768), equating to an overall correct answer percentage of 68.

## Discussion

Our study was aimed at introducing an Arabic-translated version of the Prolapse and Incontinence Knowledge Questionnaire (APIKQ) for assessing knowledge and attitudes concerning UI and POP among Arabic-speaking women. We conducted this study with a diverse sample of 300 women, ranging in age from 18 to over 65 years.

The validation of the Arabic adaptation of the PIKQ is a significant contribution, especially given its successful validations in English, Turkish, German, and Russian variants [14–17]. This establishes it as a dependable tool for evaluating patient knowledge about UI and POP in Arabic-speaking countries.

Although our study was conducted among Jordanian women, the questionnaire was translated and written in Classical Arabic, a language universally understood across the Arab world. Classical Arabic serves as the foundation for Modern Standard Arabic (MSA), making it comprehensible to speakers from all 22 Arabic-speaking countries in the Middle East and north Africa, as well as millions of Arabs worldwide. Despite regional differences in pronunciation and colloquial expressions, the fundamental grammar and vocabulary remain consistent across these diverse regions. Consequently, we believe that our translated version can be applied to Arabic-speaking countries.

To assess the validity and reliability of the Arabic version of the PIKQ, we applied various statistical measures. The questionnaire demonstrated high internal consistency, supported by Cronbach's alpha coefficients (0.824 for UI score and 0.811 for POP score), closer to the original study (0.825 and 0.895) [9] and higher than results in other adaptation studies [11]. Additionally, the item…total correlations were observed to range from 0.851 on the POP scale to 0.896 on the UI scale, suggesting a strong relationship between individual items and the overall scale.

Factor analysis, confirmed by the KMO measure of sampling adequacy as (0.637 and 0.641) and Bartlett's test of sphericity Chi-squared (66) = 197.316 (*p* < 0.001) for the UI domain and Chi-squared (66) = 183.545 (*p* < 0.001) for the POP, established the appropriateness of the questionnaire for assessing knowledge related to UI and POP. These analyses revealed four factors for the PIKQ-UI, accounting for 46.77% of the variance, and five factors for the PIKQ-POP, accounting for 54.173% of the variance. None of the items in both domains was less than 0.3, which indicates a well-defined factor structure where each item contributes meaningfully to the underlying constructs being measured; this was not the case in previous studies [15].

Test–retest reliability analysis showed consistently high correlations, with a coefficient of 0.834 for the UI scale and 0.884 for the POP scale, indicating the questionnaire's reliability over time.

In the context of pelvic floor disorders in Jordan, limited prevalence data exist. However, recent studies suggest a significant occurrence of UI among Jordanian women, with estimates ranging from 40 to 55% [18, 19]. Our findings align with this trend, as 66 out of 300 participants (22%) exhibited symptoms of incontinence. Surprisingly, only 37% of those affected had sought treatment. A possible explanation could be related to the educational backgrounds of our participants, with 156 women (52%) having attained only a high school education or less, and 36 women (12%) having received no formal education.

Education emerged as a significant factor influencing knowledge proficiency, which is consistent with previous research [3, 14, 20]. Specifically, patients with higher education levels displayed more knowledge about UI, although no significant differences were observed for POP, as shown in Table 4.

Comparisons with previous studies revealed similar trends. For instance, primiparous women and those with lower education levels were more likely to lack knowledge about UI, whereas medical field workers exhibited better UI and POP knowledge [15]. We also examined the knowledge of multiparous and nulliparous women; we did not find any significant score differences between these parity groups in the context of the POP scale. However, it is worth noting that a notable difference was observed in the UI domain.

The importance of education and seeking medical help in predicting knowledge about UI and POP emphasizes the need for targeted educational interventions and health care interactions to increase awareness and understanding of these conditions. Some studies have highlighted that the importance of women’s knowledge and perception in addition to health education about pelvic floor disorders are predictors of seeking care behavior [3–5, 14].

Regarding awareness of pelvic exercises, a substantial proportion of women in our study recognized their effectiveness in managing urinary leakage (185 women, 61.7%) and preventing prolapse progression (193 women, 64.3%), despite the absence of specific exercise details, such as the name of pelvic floor exercises (PFEs). This perspective is consistent with the findings reported by Derrar et al., wherein approximately one-third of women, comprising exclusively pregnant individuals, exhibited doubt about the benefits of pelvic floor muscle exercises [21]. Others reported that the women who were knowledge proficient in both POP and UI not only had knowledge of PFEs, but also performed Kegel exercises [22].

Regarding prolapse treatment options as examined by question numbers 9 and 11, the mean score for each was: 0.77 ± 0.422 and 0.28 ± 0.448, indicating that on average 77% and 28% of answers were correct compared with other studies that assessed knowledge using the PIKQ and found that 80% correctly answered that surgery is an option and 56% answered in favor of the pessary [3].

The strength of our study lies in its setting within an obstetrics and gynecology clinic, which enhances the applicability of the questionnaire to similar clinical environments. Additionally, our research addresses a critical public health concern, considering the documented prevalence of UI among Jordanian women and their limited tendency to seek treatment. Our study's findings have the potential to guide tailored educational initiatives and health care approaches aimed at heightening awareness and knowledge about these conditions, thereby addressing a significant health care challenge.

Furthermore, our study utilized Classical Arabic in the translated version of the PIKQ, facilitating its generalizability across Arabic-speaking regions. Despite the presence of over 25 distinct Arabic dialects derived from MSA, which shares grammatical standards with Classical Arabic but has evolved to discard certain linguistic elements, our findings remain applicable owing to the fundamental coherence of Classical Arabic across varied dialects.

One significant limitation of this study is that the diagnosis of UI and genital prolapse relied solely on patient-reported symptoms, without clinical examination data or diagnostic findings such as urodynamic studies, potentially underestimating the true prevalence of these pelvic floor disorders in our sample and not fully representing their actual prevalence in the population.

## Conclusion

The self-administered PIKQ, in its Arabic version, has been validated as a reliable tool for assessing knowledge and awareness of pelvic floor disorders among Arabic-speaking women. This tool plays a key role in closing the current knowledge gaps and correcting misconceptions about the risk factors, diagnosis, and treatment for UI and POP. Furthermore, it is essential in encouraging women to actively pursue medical care promptly.

## Abbreviations

BMI: Body mass index
CI: Confidence interval
ICC: Intraclass correlation coefficient
KMO: Kaiser–Meyer–Olkin
MSA: Modern standard Arabic
PFD: Pelvic floor dysfunction
PIKQ: Prolapse and Incontinence Knowledge Questionnaire
PIKQ-POP: Prolapse and Incontinence Knowledge Questionnaire Pelvic Organ Prolapse
PIKQ-UI: Prolapse and Incontinence Knowledge Questionnaire Urinary Incontinence
POP: Pelvic organ prolapse
SD: Standard deviation
UI: Urinary incontinence
